# Rapid Biosensor of SARS-CoV-2 Using Specific Monoclonal Antibodies Recognizing Conserved Nucleocapsid Protein Epitopes

**DOI:** 10.3390/v14020255

**Published:** 2022-01-27

**Authors:** Jong-Hwan Lee, Yujin Jung, Sung-Kyun Lee, Jung Kim, Chang-Seop Lee, Soohyun Kim, Ji-Seon Lee, Nam-Hoon Kim, Hong-Gi Kim

**Affiliations:** 1 Center for Convergent Research of Emerging Virus Infection, Korea Research Institute of Chemical Technology, Daejeon 34114, Korea; jonghwan@krict.re.kr (J.-H.L.); jyujin91@krict.re.kr (Y.J.); sunglee@krict.re.kr (S.-K.L.); jungkim@krict.re.kr (J.K.); soohyun@krict.re.kr (S.K.); ljs0102@krict.re.kr (J.-S.L.); 2 Department of Internal Medicine, Jeonbuk National University Medical School, Jeonju 54896, Jeollabuk-do, Korea; lcsmd@jbnu.ac.kr; 3 Biomedical Research Institute of Jeonbuk National University Hospital, Jeonju 54907, Jeollabuk-do, Korea

**Keywords:** COVID-19, SARS-CoV-2, virus mutation, nucleocapsid, conserved epitope, monoclonal antibody, biosensor

## Abstract

Coronavirus disease 2019 (COVID-19), the pandemic caused by severe acute respiratory syndrome coronavirus 2 (SARS-CoV-2), is characterized by symptoms such as fever, fatigue, a sore throat, diarrhea, and coughing. Although various new vaccines against COVID-19 have been developed, early diagnostics, isolation, and prevention remain important due to virus mutations resulting in rapid and high disease transmission. Amino acid substitutions in the major diagnostic target antigens of SARS-CoV-2 may lower the sensitivity for the detection of SARS-CoV-2. For this reason, we developed specific monoclonal antibodies that bind to epitope peptides as antigens for the rapid detection of SARS-CoV-2 NP. The binding affinity between antigenic peptides and monoclonal antibodies was investigated, and a sandwich pair for capture and detection was employed to develop a rapid biosensor for SARS-CoV-2 NP. The rapid biosensor, based on a monoclonal antibody pair binding to conserved epitopes of SARS-CoV-2 NP, detected cultured virus samples of SARS-CoV-2 (1.4 × 10^3^ TCID_50_/reaction) and recombinant NP (1 ng/mL). Laboratory confirmation of the rapid biosensor was performed with clinical specimens (*n* = 16) from COVID-19 patients and other pathogens. The rapid biosensor consisting of a monoclonal antibody pair (75E12 for capture and the 54G6/54G10 combination for detection) binding to conserved epitopes of SARS-CoV-2 NP could assist in the detection of SARS-CoV-2 NP under the circumstance of spreading SARS-CoV-2 variants.

## 1. Introduction

Coronavirus disease 2019 (COVID-19) is a global pandemic caused by severe acute respiratory syndrome coronavirus 2 (SARS-CoV-2) [1,2,3]. Most COVID-19 patients develop pneumonia and exhibit respiratory syndromes ranging from mild to severe, with symptoms such as fever, fatigue, a sore throat, diarrhea, and coughing [4,5,6]. Although various new RNA and DNA vaccines against COVID-19 have been developed, human-to-human transmission remains rapid and high due to virus mutations [6,7]. Reverse transcription-quantitative PCR (RT-qPCR) is considered the gold standard for diagnosis of SARS-CoV-2 [1]. However, molecular diagnosis by RT-qPCR is labor-intensive and expensive, and requires specific laboratory instruments and skilled technicians [8,9]. Molecular diagnosis by RT-qPCR is also a complicated and time-consuming procedure with an obligatory RNA isolation step [10,11].

The SARS-CoV-2 genome consists of a single positive-strand RNA molecule encoding four structural proteins: spike (S), envelope, matrix, and nucleocapsid proteins (NP) [12]. Mutations in the S protein cause amino acid substitutions in certain epitopes, some of which increase receptor-binding avidity and alter the glycosylation patterns on proteins [13,14,15]. Rahman et al. reported 1034 unique nucleotide mutations of SARS-CoV-2 NP in mutant strains (49.15%, *n* = 30,221) compared with the Wuhan reference strain [16]. These mutations in the nucleotide sequence of nucleocapsid may cause a mismatch between a target sequence and a primer–probe set. More variations than expected in the amino acid sequence of NP were observed in the RNA-binding N-terminal domain, SR-rich region, and C-terminal dimerization domain [7,16]. One specific pair of mutations of NP (R203K/G204R) influences the infectivity, fitness, and virulence of SARS-CoV-2 [17]. In RT-PCR and immunoassays, when mutations occur in regions critical for primer or antibody binding, these changes in viral nucleic acids or proteins threaten the usefulness of certain in vitro diagnostic assays [18]. Amino acid substitutions in the NP of SARS-CoV-2 might also cause low sensitivity for immunoassays such as lateral flow assays and enzyme-linked immunosorbent assays (ELISAs). Therefore, more specific antibodies binding to conserved epitopes of SARS-CoV-2 NP are required for more accurate detection by immunoassays.

In the present study, we identified conserved epitopes of SARS-CoV-2 NP and developed specific monoclonal antibodies binding to epitope peptides as antigens for rapid detection of SARS-CoV-2 NP. The binding affinity between antigenic peptides and monoclonal antibodies was examined, and a sandwich pair for the detection of SARS-CoV-2 NP was investigated. Ultimately, monoclonal antibodies binding to conserved epitopes (NP1, NP1-1, and NP4) were paired with each other for capture (75E12 and 79C12) and rapid detection (54G6 and 54G10). In the present study, the sensitivity and specificity of the rapid detection method were tested with recombinant NP of SARS-CoV-2 and clinical samples from COVID-19 patients confirmed by RT-qPCR.

## 2. Experimental Section

### 2.1. Design and Synthesis of Antigenic Peptides

**Figure 1:**
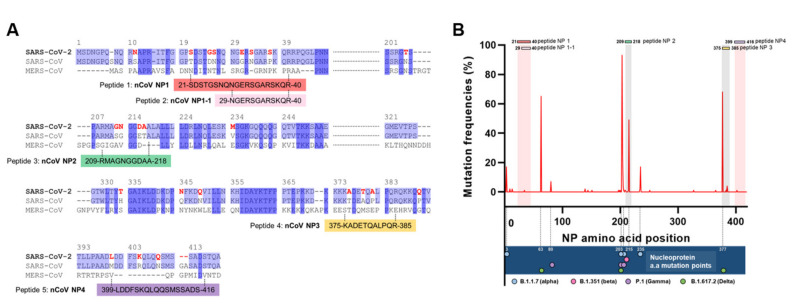
Selection of antigenic peptides for the development of SARS-CoV-2 nucleocapsid protein-specific (NP-specific) antibodies. (**A**) The amino acid sequence of SARS-CoV-2 NP is aligned with SARS-CoV NP and MERS NP. Five short peptides with high sequence specificity are selected based on the sequence alignment (NP1—red, NP1-1—pink, NP2—green, NP3—yellow, and NP4—purple). (**B**) The frequencies of amino acid substitution in SARS-CoV-2 NP in four major variants of SARS-CoV-2 (alpha, beta, gamma, and delta variants). Of the five short peptides selected by sequence alignment, the NP 1, NP 1-1, and NP 4 peptides share a highly conserved sequence despite mutation accumulation.

Amino acid sequences of SARS-CoV-2 NP (UniProtKB/Swiss-Prot: P0DTC9), SARS-CoV NP (UniProtKB/Swiss-Prot: P59595), and MERS-CoV NP (UniProtKB/Swiss-Prot: K9N4V7) were downloaded from the UniProt Knowledgebase (https://www.uniprot.org/uniprot/, accessed on 10 January 2022). The amino acid sequence of SARS-CoV-2 NP was aligned with its counterparts in SARS-CoV and MERS-CoV using commercial software (UniProt UGENE, Novosibirsk, Russia). B cell epitope prediction tools of the immune epitope database and analysis resource (National Institute of Allergy and Infectious Diseases, North Bethesda, MD, USA) were used to analyze hydrophilicity/hydrophobicity, beta-turn structure, and surface accessibility scores, respectively. The specific peptide sequences of SARS-CoV-2 NP compared to SARS-CoV NP and MERS-CoV NP were further aligned with MAFFT v7.450 [19]. Therefore, based on sequence specificity and peptide antigenicity, five different antigenic peptides were finally selected: SARS-CoV-2 NP1, N-SDSTGSNQNGERSGARSKQR-C; SARS-CoV-2 NP1-1, N-NGERSGARSKQR-C; SARS-CoV-2 NP2, N-RMAGNGGDAA-C; SARS-CoV-2 NP3, N-KADETQALPQR-C; and SARS-CoV-2 NP4, N-LDDFSKQLQQSMSSA-C (Figure 1A and Figure S1). We also investigated the frequencies of amino acid substitutions in SARS-CoV-2 NP due to viral mutations using an open-source database tool (Nextstrain, https://nextstrain.org/ncov/gisaid/global, accessed on 10 January 2022) (Figure 1B). The five antigenic peptides were synthesized by Abclon (Seoul, Korea), and analyzed by high-performance liquid chromatography and mass spectrometry.

### 2.2. Immunization and Antibody Production

Synthetic peptide antigens (100 μg) were mixed with Freund’s adjuvant (Sigma-Aldrich, St. Louis, MO, USA, cat. no. F5506) and intraperitoneally injected into BALB/c mice (DBL Co., Ltd., Eumseong, Korea). After 2 weeks, 100 μg of each antigen was diluted in phosphate-buffered saline (PBS), injected again, and 3 days thereafter the mouse spleen was removed and lymphocytes were isolated. The isolated lymphocytes were mixed with the myeloma cell line SP2/0-Ag14 (ATCC, cat. no. CRL-1581) at a 5:1 ratio and fused using polyethylene glycol (PEG)-1500 (Roche, Basel, Switzerland, cat. no. 783641). The fused cells were cultured in medium containing HAT supplement (Sigma-Aldrich, cat. no. H0262), and fused cells (hybridomas) were selectively sorted out and cultured. The resulting hybridoma cells were examined by ELISA to determine whether they were producing antibodies that bind antigens. Each SARS-CoV-2 NP peptide-Fc or ChromPure human IgG (hIgG, Jackson ImmunoResearch Laboratories Inc., West Grove, PA, USA, cat. no. 009-000-003) was immobilized at room temperature on a 96-well plate (Corning Inc., Corning, NY, USA, cat. no. 3590) at a concentration of 1 μg/mL for 1 h. The plate was washed three times with 0.05% Triton X-100 (TBS-T) and blocked at room temperature with 300 mL of 2% skim milk (TBS-T/SM) for 30 min. The blocked plate was washed three times, hybridoma culture broth was added, and antibodies were allowed to bind at 37 °C for 1 h. After washing three times, the secondary antibody anti-mouse IgG-HRP (Pierce, Appleton, WI, USA, cat. no. 31439) was diluted in TBS-T/SM at a 1:5000 ratio and allowed to bind at 37 °C for 1 h. After washing three times, TMB (Surmodics, Eden Prairie, MN, USA, cat. no. TMBC-1000-01) was added, color was developed at room temperature for 5 min, and 1 M sulfuric acid (DukSan, Ansan-si, Korea, cat. no. 254) was added to stop the color development. The absorbance was measured at 450 nm using a Victor X3 instrument (PerkinElmer, Waltham, MA, USA, cat. no. 2030-0030), and antibodies that bind specifically to SARS-CoV-2 NP peptides were selected.

### 2.3. Biolayer Interferometry (BLI)

The binding affinities between the SARS-CoV-2 NP antigen and monoclonal antibodies (seven different monoclonal antibodies: 54F10, 54G6, 54G10, 54H2, 66E10, 75E12, and 79C12) were analyzed by BLI using a BLITz instrument (ForteBio, Fremont, CA, USA). Polyhistidine-tagged recombinant SARS-CoV-2 NP antigen (cat. no. 40588-V08B) was purchased from Sino Biological (Beijing, China). BLI was initiated by hydrating nickel-coated NTA biosensors (ForteBio, part no. 18-5102) for 10 min. After hydration, BLI was performed over five steps: initial baseline (20 s), antigen immobilization (300 s), second baseline (120 s), antibody binding (300 s), and dissociation (300 s). Baseline and dissociation steps were performed with sample dilution buffer comprising 0.02% Tween 20, 150 mM NaCl, and 1 mg/mL bovine serum albumin (BSA) in 10 mM PBS with 0.05% sodium azide (pH 7.4). Polyhistidine-tagged SARS-CoV-2 NP antigen (100 µg/mL) was first loaded onto the NTA biosensor surface for antigen immobilization. After removing residual antigen in the second baseline step, antibody was loaded to evaluate the specific interaction between antigen and antibody. Four different concentrates (100 µg/mL, 50 µg/mL, 25 µg/mL, and 10 µg/mL) of antibody were used to analyze the effect of antibody concentration on the association–dissociation pattern. Binding constants were calculated from the resulting four association–dissociation curves based on a 1:1 binding model.

### 2.4. Fabrication of the Lateral Flow Immunoassay (LFIA) Device

**Scheme 1:**
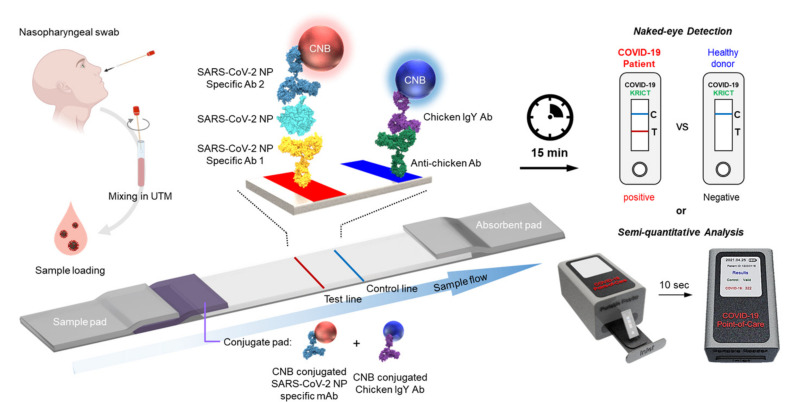
Detection of the SARS-CoV-2 NP antigen with lateral flow immunoassay (LFIA). The LFIA consists of a sample pad, conjugate pad, nitrocellulose membrane, and absorbent pad. The conjugate pad contains a CNB-conjugated SARS-CoV2 NP-specific antibody and a chicken IgY antibody, used as signal molecules for the test line and control line, respectively. SARS-CoV-2 NP antigen extracted from a patient’s nasopharyngeal swab is loaded into the LFIA, and the sample flows through the LFIA via capillary force. After 15 min of sample loading, SARS-CoV-2 NP is detected in the test line, showing a red line. Detection of the SARS-CoV-2 NP antigen can be confirmed visually or semi-quantitatively.

The LFIA device consisted of a sample pad (Ahlstrom, Helsinki, Finland), a conjugate pad (Ahlstrom), a nitrocellulose membrane (PALL Corporation, New York, NY, USA), and an absorbent pad (Ahlstrom), as shown in Scheme 1. Capture antibody (66E10, 75E12, and 79C12) and anti-chicken IgY antibody (Bore Da Biotech, Seongnam, Korea, cat. no. 23000) were used for the test line and control line, respectively. Capture antibody (0.5 mg/mL) and anti-chicken IgY antibody (0.5 mg/mL) were immobilized onto the nitrocellulose membrane using a line dispenser (BTM Inc., Uiwang, Korea) at a dispensing speed of 50 mm/s and a dispensing rate of 0.5 μL/cm. After drying for 1 h at 37 °C in a vacuum oven (FDU-1200, EYELA, Tokyo, Japan; JSVO-30T, JSR, Gongju, Korea), the nitrocellulose membrane was placed in blocking solution (10 mM 2-amino-2-methyl-1-propanol pH 9.0, 0.5% BSA, 0.5% β-lactose, 0.05% Triton X-100, 0.05% sodium azide) and further incubated in the vacuum oven (37 °C). After washing three times with sodium phosphate buffer (5 mM, pH 7.5), the nitrocellulose membrane was dried in the oven at 37 °C for 1 h and stored in a desiccator.

The conjugate pad contained a signal molecule displaying a unique color for target detection. Two different colored cellulose nanobeads (CNBs) were purchased from Asahi Kasei Fibers Corporation (NanoAct, Miyazaki, Japan, cat. no. RE2AA[red]/BL1AA[blue]), and used as signal molecules. Antibody conjugation to CNBs was performed with a CNB Conjugation Kit (DCN Diagnostics, Carlsbad, CA, USA, cat. no. CKNB-010) following the manufacturer’s instructions. A detailed conjugation method was presented in our previous works [9,12]. The detection antibodies 54G6 and 54G10 were employed for detecting the NP antigen and forming a sandwich complex with the capture antibody at the test line. To improve the detection performance, we used a 4:1 (*v*/*v*) mixture of 54G10 labeled with CNB and 54G6 labeled with CNB. Each component of the LFIA strip was precisely assembled and cut to a 38 mm width, followed by integration into the housing.

### 2.5. Screening of Sandwich Pairs for Detecting the SARS-CoV-2 NP Antigen

Forty-two sandwich pairs obtained from seven monoclonal antibodies (54F10, 54G6, 54G10, 54H2, 66E10, 79C12, and 75E12) were tested to identify the optimal pair for detecting the SARS-CoV-2 NP antigen. Each monoclonal antibody was applied to capture (immobilized onto the nitrocellulose membrane, P_C_) and detection (labeled with CNB, P_D_) probes. SARS-CoV-2 NP (cat. no. 40588-V08B), SARS-CoV NP (cat. no. 40143-V08B), and MERS-CoV NP (cat. no. 40068V08B) were purchased from Sino Biological (Beijing, China). SARS-CoV NP and MERS-CoV NP were used as negative controls for specificity evaluation of the sandwich pairs. Each target antigen (50 ng) was spiked into sample running buffer (Borex, 5 mM EDTA, 200 mM urea, 1% Triton X-100, 0.5% Tween 20, 500 mM NaCl, 1% PEG-200), and a 100 µL sample containing antigen was loaded onto the LFIA device. After 15 min of sample loading, the test lines were photographed with a smartphone, and their intensities were measured by a Light G portable line analyzer (Wells Bio, Seoul, Korea). The intensity values of more than 50 were validated as positive by the manufacturer’s recommendation of the portable analyzer.

The mixing ratio for P_D_ was further optimized to improve the detection sensitivity. Five different mixing ratios, 54G6(10):54G10(0), 54G6(8):54G10(2), 54G6(5):54G10(5), 54G6(2):54G10(8), and 54G6(0):54G10(10), were tested, and the 54G6(2):54G10(8) ratio was selected. Optimization was evaluated under three conditions (buffer only, 50 ng SARS-CoV-2 NP, and inactivated SARS-CoV-2 at 2.8 × 10^4^ TCID_50_), and 79C12 monoclonal antibody was used as P_C_. The three optimal pairs achieving the best performance were eventually selected: Pair 1, 75E12(P_C_)–54G6(2)/54G10(8)(P_D_); Pair 2, 79C12(P_C_)–54G6(2)/54G10(8)(P_D_); Pair 3, 66E10(P_C_)–54G6(2)/54G10(8)(P_D_).

### 2.6. LFIA Limit of Detection (LOD) Determination Using Recombinant Antigens and Viral Samples

To analyze the sensitivity of the LFIA based on the selected pairs, serially diluted recombinant SARS-CoV-2 NP antigens and γ-irradiated inactivated SARS-CoV-2 (BEI Resources, Manassas, VA, USA, cat. no. NR-52287) were used. The final concentrations of diluted SARS-CoV-2 NP antigens and inactivated SARS-CoV-2 ranged from 500 ng/mL to 200 pg/mL, and 2.8 × 10^4^ TCID_50_/reaction to 1.4 × 10^3^ TCID_50_/reaction, respectively. The diluted samples were mixed with running buffer (Borex, 5 mM EDTA, 200 mM urea, 1% Triton X-100, 0.5% Tween 20, 500 mM NaCl, 1% PEG-200) at a ratio of 1:9 (*v*/*v*). Running buffer (100 μL) containing different concentrations of the target was added to the LFIA device. After 15 min, LFIA strips were photographed, and the intensities of the test lines were measured by a Light G line analyzer. The test and control lines were further analyzed by a Sapphire Biomolecular imager (Azure Biosystems, Dublin, CA, USA) to confirm the signal accuracy.

### 2.7. Laboratory Confirmation Using Clinical Samples

Nasopharyngeal swab samples in universal transport media (UTM) from COVID-19 patients were kindly provided by Chonbuk National University Hospital (Korea). Clinical specimens from COVID-19 patients were collected according to a registration protocol approved by the Institutional Review Board (IRB) of Chonbuk National University Hospital. All patients provided written informed consent (IRB registration number: CUH2021-06-036-002). Nasopharyngeal swabs from healthy donors were purchased from Lee Biosolutions (Maryland Heights, MO, USA, cat. no. 991-31-NC) and suspended in UTM (NobleBio, Hwaseong-si, Korea, cat. no. UTNFS-3B-1). Detailed information on COVID-19 patients and healthy donors is provided in Table S1. Swab samples from patients and donors were mixed with LFIA running buffer at a 1:1 (*v*/*v*) ratio, incubated for 10 min at room temperature, and 100 μL of the mixture was used for clinical assessment of the LFIA device. After 15 min of sample loading, the results of COVID-19 infection were confirmed with the naked eye, and the intensities of the test lines were further analyzed with a portable analyzer. In addition, quantitative reverse transcription polymerase chain reaction (RT-qPCR) was performed to compare diagnostic performance with the LFIA device. Using a specific primer–probe set (forward primer: 5′-AAATTTTGGGGACCAGGAAC-3′; reverse primer: 5′-TGGCAGCTGTGTAGGTCAAC-3′; TaqMan probe: 5′ [FAM]ATGTCGCGCATTGGCATGGA[BHQ1] 3′), which had been confirmed in our previous study [1], the SARS-CoV-2-specific NP gene (N gene) was amplified and detected by reverse transcription (55 °C for 10 min) and amplification for 45 cycles (95 °C for 10 s, 60 °C for 30 s). The results of RT-qPCR (C_t_ value and real-time amplification curves) were analyzed using a CFX 96 Touch Real-Time PCR System (Bio-Rad, Hercules, CA, USA). Quantification of the viral load in the clinical samples was performed using a SARS-CoV-2 RNA standard purchased from the Korea Research Institute of Standards and Science (KRISS, Daejeon, Korea, cat. no. 111-10-506).

Furthermore, specificity analysis of the LFIA device was performed with human coronaviruses and other respiratory pathogens. Human coronavirus OC43 (OC43, ATCC No. VR-1558), human coronavirus 229E (229E, ATCC No. VR-740), human parainfluenza virus 1 (HPIV-1, ATCC No. VR-94), human parainfluenza virus 3 (HPIV-3, ATCC No. VR-93), human adenovirus 7a (Adeno 7a, ATCC No. VR-848), human rhinovirus 1B (Rhinovirus 1b, ATCC No. VR-1645), human respiratory syncytial virus (RSV, ATCC No. VR-1580), and *Mycobacterium tuberculosis* (MTB, ATCC No. 25177) were purchased from ATCC (Manassas, VA, USA). Human influenza A virus H1N1 (Influenza A, KBPV-VR-33) and human influenza B virus (Influenza B, KBPV-VR-34) were purchased from the Korea Bank for Pathogenic Viruses (Seoul, Korea). Control virus samples were diluted to 10^6^ TCID_50_/reaction (OC43 was diluted to 5 × 10^5^ TCID_50_/reaction) in running buffer. The MTB sample was diluted twofold with running buffer and used for specificity testing (the exact concentration of MTB is unknown).

## 3. Results and Discussion

### 3.1. Development of SARS-CoV-2 NP-Specific Monoclonal Antibodies Derived from Short Conserved Peptides in the NP Antigen

The NP is a viral antigen that plays a crucial role in packaging viral genome RNA (Figure S2). The SARS-CoV-2 NP is abundantly expressed during the viral infection and is highly immunogenic [20,21,22]. Therefore, it is considered an ideal target for a diagnostic antigen of SARS-CoV-2 [21,22,23,24]. However, SARS-CoV-2 NP shares sequence homology with NPs from other coronaviruses (~90% identity with SARS-CoV NP and ~50% identity with MERS-CoV NP; Figure S1). The sequence homology of antigen proteins might lead to cross-reactivity and specificity issues with other CoV NPs. To overcome these shortcomings, we explored specific short peptides of SARS-CoV-2 NP with sequences that differed from SARS-CoV NP and MERS NP as antigenic determinants of antibody production (Figure 1A): NP 1 comprising amino acid (aa) residues 21–40, NP 1-1 (aa 29–40), NP 2 (aa 209–218), NP 3 (aa 375–385), and NP 4 (aa 399–416). Furthermore, three peptides (NP 1, NP 1-1, and NP 4) showed highly conserved sequences under mutations accumulated in SARS-CoV-2 NP (Figure 1B).

**Scheme 2:**
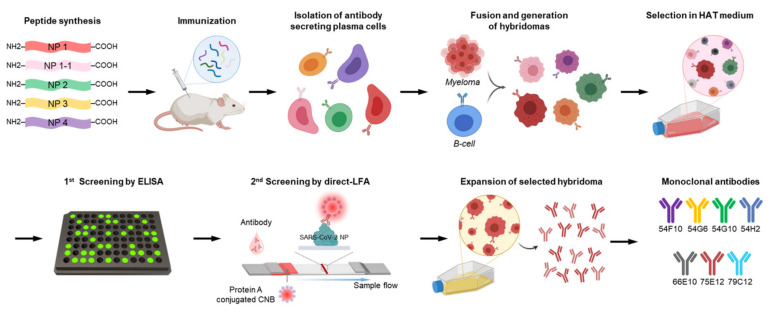
Overall scheme of SARS-CoV-2 NP-specific antibody production. After immunizing mice with each of the five selected peptides (NP 1, NP 1-1, NP 2, NP3, and NP 4), antibody-secreting plasma cells were isolated and fused with myeloma to generate hybridomas. The hybridoma cells were selectively cultured and screened by ELISA (1st) and direct-LFA (2nd). Selected hybridoma cells were injected into the mouse abdominal cavity. After 14 days, produced antibodies were purified by affinity chromatography. Seven monoclonal antibodies (54F10, 54G6, 54G10, 54H2, 66E10, 79C12, and 75E12) were eventually obtained.

Scheme 2 shows the overall scheme of SARS-CoV-2 NP-specific antibody production. Briefly, after immunizing mice with each of the selected peptides (NP 1, NP 1-1, NP 2, NP 3, NP4), serum was collected, and antibody titers were confirmed by ELISA. Spleen tissue was extracted from mice immunized with peptide antigen, B lymphocytes were isolated, and cell fusion was performed with myeloma. The resulting hybridoma cells were selectively cultured in HAT media for 2 weeks. Through ELISA using a culture medium, primary screening was performed to determine whether the hybridomas produced antibodies that properly bind to the target antigens. Moreover, culture medium containing specific antibodies was further analyzed by indirect LFIA. Three different NPs from SARS-CoV-2, SARS-CoV, and MERS-CoV were pre-immobilized onto a nitrocellulose membrane, and the binding affinities against the produced antibodies were evaluated (Figure S3). The specific clones (54F10, 54G6, 54G10, 54H2, 66E10, and 79C12) displaying high affinity for SARS-CoV-2 NP and no binding to other NPs were selected for in vivo antibody production (75E12 was added later). Selected hybridoma cells were injected into the mouse abdominal cavity. After 14 days, ascites were collected from the abdominal cavity, and the antibody was purified by affinity chromatography. Seven different monoclonal antibodies (54F10, 54G6, 54G10, 54H2, 66E10, 79C12, and 75E12) with high sensitivity and specificity to SARS-CoV-2 NP were eventually obtained (detailed methods for the developing monoclonal antibodies were displayed in Experimental Section 2.2).

### 3.2. Evaluation of the Binding Affinity of Monoclonal Antibodies against the SARS-CoV-2 NP Antigen

**Figure 2:**
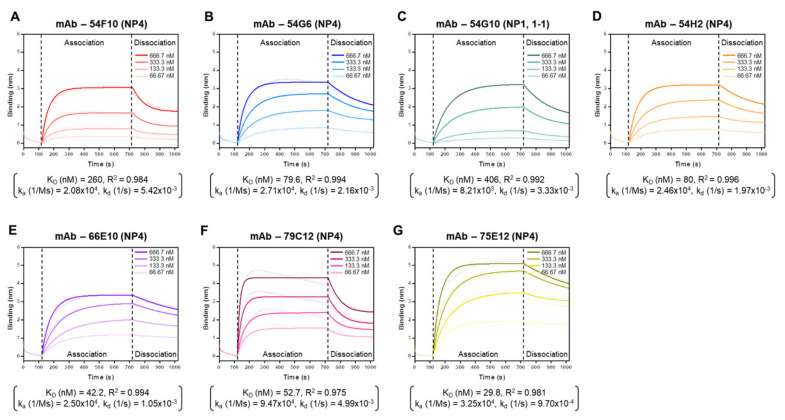
Biolayer interferometry (BLI) results of monoclonal antibodies against the SARS-CoV-2 NP antigen. Association and dissociation curves resulting from the antibodies–NP-antigen binding events are obtained using the BLI technique. Real-time binding sensorgrams are represented in dotted lines and their fitting curves are represented in solid lines: (**A**) 54F10 (red), (**B**) 54G6 (blue), (**C**) 54G10 (green), (**D**) 54H2 (orange), (**E**) 66E10 (purple), (**F**) 79C12 (pink), (**G**) 75E12 (yellow). The 54F10, 54G6, 54H2, 66E10, 79C12, and 75E12 monoclonal antibodies are produced from NP4 peptide as an antigenic determinant, and 54G10 is derived from the antigenic peptide NP1 or NP 1-1. Four different concentrates of antibody are used to analyze the association–dissociation pattern and the binding constants are calculated from the resulting fitting curves based on a 1:1 binding model.

Specific interactions between the developed monoclonal antibodies and SARS-CoV-2 NP antigen were investigated using BLI, which measures biomolecular interactions based on differences in the interference pattern resulting from binding events. Polyhistidine-tagged SARS-CoV-2 NP antigen was first immobilized on the NTA biosensor tip through specific interactions between nickel and histidine residues. Next, four different concentrations of antibodies were applied to the pre-immobilized SARS-CoV-2 NP antigen, and association and dissociation curves resulting from the antigen–antibody binding events were obtained (detailed methods for measuring the binding affinity using the BLI were displayed in Experimental Section 2.3). Representative real-time binding sensorgrams (dotted lines) and fitting curves (solid lines) are shown in Figure 2. Binding constants were calculated from the fitting curves based on a 1:1 binding model. The K_D_ values of monoclonal antibodies against the SARS-CoV-2 NP antigen ranged from tens to hundreds of nM (mAb (K_D_ value against NP), 54F10 = 260 nM, 54G6 = 79.6 nM, 54G10 = 406 nM, 54H2 = 80 nM, 66E10 = 42.2 nM, 79C12 = 52.7 nM, and 75E12 = 29.8 nM), indicating comparable K_D_ values to commercially available antibodies. This suggests that newly developed antibodies with K_D_ values in the nanomolar range are capable of detecting SARS-CoV-2-specific antigens.

The recognition site of each antibody for its target antigen depends on the antigenic epitope used for immunization to produce the antibody. In this study, monoclonal antibodies were produced by immunization with a short antigenic peptide derived from the target antigen. The antibodies produced in this way bind to the antigen by recognizing a specific region of the antigen rather than the entire antigen region. Therefore, it can be conferred with specificity for the NP of other coronaviruses and sensitivity to mutations accumulated in the NP of SARS-CoV-2. To verify the recognition site of each antibody, binding affinities between antibodies and their antigenic peptides were determined by BLI. As shown in Figure S4A, biotinylated antigenic epitopes were immobilized onto a streptavidin-coated BLI biosensor tip, and four concentrations of each monoclonal antibody were applied to obtain association and dissociation curves. Monoclonal antibody 54G10 was derived from the antigenic epitope NP 1 or NP 1-1. Meanwhile, 54F10, 54G6, 54H2, 66E10, 79C12, and 75E12 were produced from the NP 4 epitope. K_D_ values of 54G10 antibody for NP 1 and NP 1-1 were 46 nM and 26.2 nM, respectively, indicating that 54G10 antibody binds to the NP 1 (or NP 1-1) sequence with high affinity (Figure S4B,C). Furthermore, since the binding affinity for NP 1-1 was slightly higher than that for NP 1, we assumed that the recognition site of 54G10 is mainly located on the NP 1-1 side. By contrast, other antibodies (54F10, 54G6, 54H2, 66E10, 79C12, and 75E12) showed high affinities for the NP 4 peptide (K_D_ values of 54F10, 54G6, 54H2, 66E10, 79C12, and 75E12 were 64.4 nM, 0.65 nM, 4.22 nM, 19.5 nM, 32.1 nM, and 3.66 nM, respectively), which means that these antibodies recognized short sequences of NP 4 to which they bound strongly (Figure S4D). The seven monoclonal antibodies developed in our study selectively recognized specific short sequences on the SARS-CoV-2 NP antigen. These specific sequences were differentiated from the NPs of other coronaviruses, and conserved sequences were not affected by the mutation. As we previously mentioned, the changes of protein sequences resulting from viral mutations lead to low sensitivity in the diagnosis [18]. If SARS-CoV-2 variants spread rapidly, developing an antibody that recognizes a specific peptide sequence rather than the entire antigen might be more effective for diagnosing COVID-19.

### 3.3. Identification of the Optimal Sandwich Pair for Detecting SARS-CoV-2 NP Antigens

**Figure 3:**
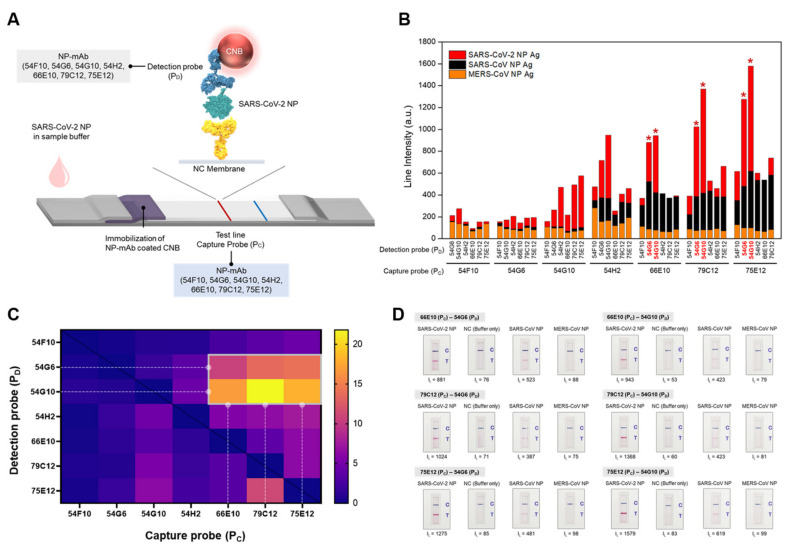
Discovery of the optimal sandwich pair for detecting SARS-CoV-2 NP antigen. (**A**) Schematic illustration of the LFIA consisting of seven kinds of capture and detection probes. A total of 42 pairs obtained from seven kinds of monoclonal antibodies are evaluated. (**B**) Overlapped bar graph showing line intensities in the test line according to each sandwich pair and target antigen. Each pair is assessed for detection sensitivity and specificity with 50 ng of NP antigen from SARS-CoV-2 (red), SARS-CoV (black), and MERS-CoV (orange) (P_C_: capture probe; P_D_: detection probe). Red marks above the bars and detection probes indicate optimal pairs selected for SARS-CoV-2 NP detection. (**C**) Heat-map for the normalized line intensities of each sandwich pair. The line intensities are normalized for the absence of the target antigen. Six pairs (marks in gray box) are finally selected for the sandwich pairs for detecting the SARS-CoV-2 NP antigen. (**D**) Representative images of the LFIA results for each of the selected six pairs (I_L_: line intensity of the test line measured by a portable line analyzer).

For the sensitive and selective detection of the target antigen via LFIA, a specific antibody pair that recognizes different regions of the target antigen is required. The selection of the optimum pair for the target antigen is a prerequisite for improving the diagnostic performance of the LFIA. To discover the optimum pair for detecting SARS-CoV-2 NP, the diagnostic performance of 42 pairs obtained from seven kinds of monoclonal antibodies was assessed (Figure 3A). Each monoclonal antibody was applied to capture (immobilized onto the nitrocellulose membrane, P_C_) and detection (labeled with CNB, P_D_) probes, and six different P_D_ were paired with a P_C_ that was not used as a P_D_. SARS-CoV-2 NP (50 ng), SARS-CoV NP (50 ng), and MERS-CoV NP (50 ng) were used as target antigens to evaluate the sensitivity and specificity of these pairs. As shown in Figure 3B, SARS-CoV-2 NP was successfully detected by many pairs, and when 75E12, 79C12, 66E10, and 54H2 (in order of intensity) were used as P_C_, the line intensities were increased overall. Moreover, 54G6 and 54G10 were best suited for use as P_D_. When using the same P_C_, 54G10 as P_D_ displayed the highest line intensity in all cases (followed by 54G6). As mentioned above, 54G10 has a different recognition site (NP 1 or NP 1-1) from other antibodies (NP 4). Therefore, 54G10 likely forms sandwich complexes with other antibodies more efficiently. In addition, we can speculate that the recognition site of 54G6 does not overlap with other antibodies used as P_C_, even if it is produced using the same antigenic epitope (NP 4).

On the other hand, there was no cross-reactivity when detecting MERS-CoV NP, but a weak false-positive signal was observed when detecting SARS-CoV NP. Although short, specific peptides were used for antibody production as antigenic determinants, it was not possible to distinguish SARS-CoV-2 NP from SARS-CoV NP completely, because the homology between the two viruses is so high (~90%). Since SARS-CoV is not currently believed to be circulating, a weak false-positive signal is not a significant concern at this time. At the end of this study, the specificity of LFIA was further confirmed by cross-reactivity analysis with other control viruses that can spread and exhibit symptoms similar to those of SARS-CoV-2.

The line intensities at the baseline level (absence of target) are different for each pair, as interactions between the capture and detection probes can lead to false-positive signals. Therefore, the line intensities were normalized for the absence of target antigen to accurately assess the detection sensitivity of the pair (Figure 3C). Six pairs, namely 79C12(P_C_)–54G10(P_D_), 79C12(P_C_)–54G6(P_D_), 75E12(P_C_)–54G10(P_D_), 75E12(P_C_)–54G6(P_D_), 66E10(P_C_)–54G10(P_D_), and 66E10(P_C_)–54G6(P_D_), displayed remarkable detection sensitivity and were selected as sandwich pairs for detecting SARS-CoV-2. Among these, the 79C12(P_C_)–54G10(P_D_) pair exhibited the highest line intensity, followed by 75E12(P_C_)–54G10(P_D_). This suggests that 79C12 and 75E12 are most effective as capture probes, while 54G10 is most suitable as a detection probe. Representative images of the LFIA results for each of the six pairs are shown in Figure 3D and Figure S5.

### 3.4. Analysis of LFIA Sensitivity

LFIAs for each selected pair were further optimized to increase detection sensitivity and reduce false-negative signals. The false-negative signal was reduced by adjusting the buffer composition, blocking solution, and concentration of the detection probe. This eliminated false-negative signals, and gave line intensity values <50 (the final optimized experimental conditions are described in the Methods). Meanwhile, the detection probes were optimized by mixing the selected detection probes 54G6 and 54G10 to improve detection sensitivity. Simultaneous use of these two antibodies as detection probes can enhance the detection signal because the recognition sites of the antibodies are different from each other. We confirmed the change in detection performance according to the mixing ratio of 54G6 and 54G10. The detecting performance was evaluated with 50 ng of recombinant SARS-CoV-2 antigen and 2.8 × 10^4^ TCID_50_ of inactivated viral samples, and five different mixing ratios were tested (Figure S6). When the mixing ratio of 54G6 and 54G10 was 2:8 (*v*/*v*), the line intensity was the highest for both recombinant antigen and virus samples. Therefore, a mixture of 54G6 and 54G10 at a 2:8 (*v*/*v*) ratio was used as a detection probe for subsequent sensitivity analysis and clinical evaluation.

**Figure 4:**
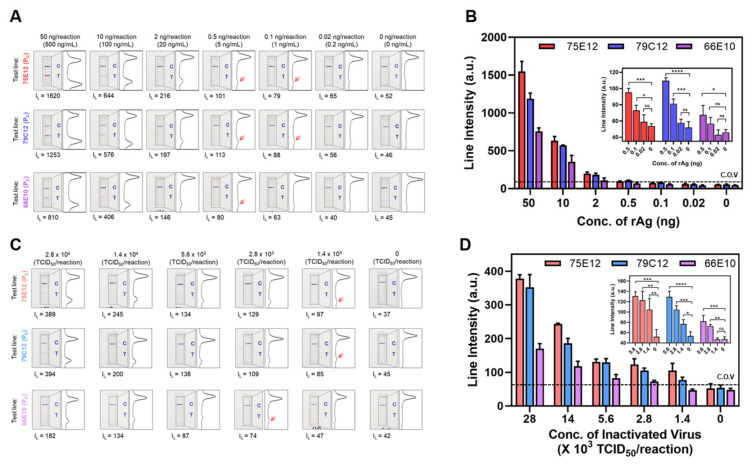
Sensitivity analysis of the optimal sandwich pairs. (**A**) Results for the detection sensitivity of the selected pairs with recombinant SARS-CoV-2 NP antigen. Three pairs of LFIAs; <Pair 1> 75E12(P_C_)–54G6/54G10(P_D_), <Pair 2> 79C12(P_C_)–54G6/54G10(P_D_), and <Pair 3> 66E10(P_C_)–54G6/54G10(P_D_), are tested with the serially diluted recombinant NP antigens (concentration ranges: 50 ng/reaction to 20 pg/reaction). The LFIA strips are photographed and the intensity of the test line is measured using a portable line analyzer (I_L_: line intensity). Furthermore, the intensities of the test and control lines are converted to a peak histogram. (**B**) Bar graph showing the sensitivity analysis results for the selected pairs; Pair 1 (red), Pair 2 (blue), and Pair 3 (purple). Inset) The detection intensity in the low concentration range (0.5 ng to 0.02 ng antigen) (*p*-values: ns > 0.05, * *p* ≤ 0.05, ** *p* ≤ 0.01, *** *p* ≤ 0.001, **** *p* ≤ 0.0001). (**C**) Results for the detection sensitivity of the selected pairs with serial diluted viral samples (concentration ranges: 2.8 × 10^4^ TCID_50_ to 1.4 × 10^3^ TCID_50_). (**D**) Bar graph showing the detection sensitivity of the viral sample. Inset) The detection intensity in the low concentration range (5.6 × 10^3^ TCID_50_ to 1.4 × 10^3^ TCID_50_ of SARS-CoV-2) (*p*-values: ns > 0.05, * *p* ≤ 0.05, ** *p* ≤ 0.01, *** *p* ≤ 0.001, **** *p* ≤ 0.0001). The cutoff value (C.O.V.) is determined as the mean value of the line intensities in the absence of antigen plus three times the standard deviation.

The sensitivity of the LFIAs with selected pairs was evaluated with recombinant SARS-CoV-2 NP antigens and inactivated SARS-CoV-2 viral samples. Three pairs of LFIAs, <Pair 1> 75E12(P_C_)–54G6/54G10(P_D_), <Pair 2> 79C12(P_C_)–54G6/54G10(P_D_), and <Pair 3> 66E10(P_C_)–54G6/54G10(P_D_), were tested using serially diluted samples (concentration range for recombinant antigen = 50 ng/reaction to 20 pg/reaction, and for inactivated viral sample = 2.8 × 10^4^ TCID_50_ to 1.4 × 10^3^ TCID_50_). At 15 min after sample loading into the LFIA device, test and control lines were photographed with a smartphone and analyzed with a portable line analyzer to measure the line intensity, and an image analyzer was used to convert the line intensity into signal peaks (Figure 4 and Figure S7). The line intensity gradually decreased as the dilution factor increased, the antigen (or virus) concentration decreased, and there were no false-positive signals in the absence of antigen (or virus). The LOD in Figure 4B,D was calculated as the mean value of the negative control plus three times the standard deviation. In the case of Pair 1, the SARS-CoV-2 NP antigen and the inactivated viral sample were successfully detected even at low concentrations (NP antigen = 100 pg/reaction; viral sample = 1.4 × 10^3^ TCID_50_/reaction). Pair 2 showed similar sensitivity to Pair 1 for both antigen detection and virus detection. However, the sensitivity of Pair 3 was slightly lower than that of Pair 1 and Pair 2. The LFIA composed of Pair 3 could detect up to 500 pg of recombinant antigen and 2.8 × 10^3^ TCID_50_ of virus. Therefore, two types of LFIA consisting of Pair 1 or Pair 2 were applied for clinical evaluation and cross-reactivity testing.

### 3.5. LFIA Clinical Assessment

**Figure 5:**
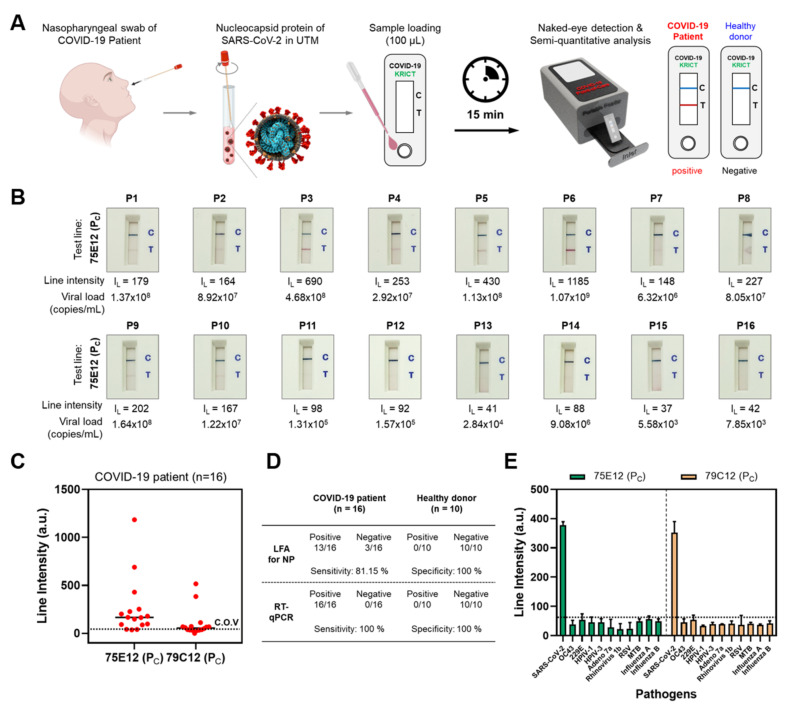
Clinical assessment of the LIFA. (**A**) Scheme of the laboratory confirmation procedure of the LFIA with newly developed sandwich pairs. Nasopharyngeal swabs from COVID-19 patients are placed into the UTM and mixed with running buffer in a 1:1 (*v*/*v*) ratio. An amount of 100 μL of the mixed solution is loaded into the LFIA, and, 15 min later, the presence of SARS-CoV-2 NP antigen in the clinical sample is confirmed by visual and portable line analyzer. (**B**) Detection sensitivity results of Pair 1-based LFIA with clinical specimens. Nasopharyngeal swabs from COVID-19 patients (*n* = 16) are applied to the LIFA device. After 15 min of sample loading, the results of COVID-19 infection are confirmed with the naked eye, and the intensities of the test lines are further analyzed with a portable analyzer (I_L_: line intensity). RT-qPCR quantifies the viral load of the COVID-19 patient specimens. (**C**) Dot graph of clinical assessment results for Pair 1- and Pair 2-based LFIA. The cut-off-value (C.O.V.) is determined as the mean value of the line intensities of healthy donors plus three times the standard deviation. (**D**) Laboratory confirmation results of the Pair 1-based LFIA compared to the RT-qPCR using clinical samples. Nasopharyngeal swab samples from COVID-19 patients (*n* = 16) and healthy donors (*n* = 10) are applied to the LFIA and RT-qPCR. RT-qPCR is performed with specific primer–probe sets for detecting the SARS-CoV-2-specific gene (N gene), and viral load in the clinical sample is investigated with the standard curve of N-gene amplicon obtained from standard SARS-CoV-2 RNA. (The detailed information of the primer–probe set is presented in Experimental Section 2.7). (**E**) Bar graph of specificity analysis with human coronaviruses (OC32 and 229E) and other respiratory pathogens such as human parainfluenza virus 1 (HPIV-1), human parainfluenza virus 3 (HPIV-3), human adenovirus 7a (Adeno 7a), human rhinovirus 1B (Rhinovirus 1b), human respiratory syncytial virus (RSV), and Mycobacterium tuberculosis (MTB). The concentration of the control virus sample is 10^6^ TCID_50_/reaction (excluding OC43: 5 × 10^5^ TCID_50_/reaction). Green bar: Pair 1-based LFIA, yellow bar: Pair-2 based LFIA.

Figure 5A illustrates the procedure for laboratory confirmation of LFIAs with newly developed SARS-CoV-2 NP-specific monoclonal antibodies. The previously selected <Pair 1> 75E12(P_C_)–54G6/54G10(P_D_) and <Pair 2> 79C12(P_C_)–54G6/54G10(P_D_) were applied in LFIAs, and the clinical assessment was evaluated. Nasopharyngeal swabs from COVID-19 patients were thoroughly mixed in UTM, and UTM containing SARS-CoV-2 NP antigen was loaded into the LFIA. At 15 min after loading the samples, the clinical specimens were confirmed for the presence of SARS-CoV-2 NP antigen (i.e., SARS-CoV-2 infection could be identified) by the naked eye (qualitative) and by a portable line analyzer (semi-quantitative). Sixteen nasopharyngeal swabs from COVID-19 patients and ten nasopharyngeal swabs from healthy donors were applied to the LFIA device. (The information of the clinical specimens was presented in Experimental Section 2.7 and Table S1). The LFIA composed of Pair 1 successfully detected the SARS-CoV-2 NP antigen in thirteen of sixteen clinical samples from COVID-19 patients (sensitivity: 81.15%, Figure 5B–D and Figure S8). However, while the Pair 2-based LFIA detected eight clinical specimens, it failed to detect eight patient specimens (patient no. 7, 10, 11, 12, 13, 14, 15, and 16; Figure S9). Moreover, the Pair 1-based LFIA exhibited higher line intensities than the Pair 2-based LFIA for the same clinical specimens, indicating that the sensitivity of the Pair 1-based LFIA was higher. By contrast, there were no false-positive signals for the specimens from ten healthy donors for Pair 1- or Pair 2-based LFIAs (Figure S10).

RT-qPCR analysis was also performed on nasopharyngeal swabs from COVID-19 patients to accurately measure the viral load in clinical specimens. Viral load in clinical samples was investigated using a standard curve of the N-gene amplicon obtained from a standard SARS-CoV-2 RNA. The viral loads in the clinical specimens ranged from 1.07 × 10^9^ copies/mL to 5.58 × 10^3^ copies/mL, and all healthy donor samples were identified as virus-negative (Figure 5B,D and Figure S11). Specifically, the viral loads in patient specimens 13, 15, and 16, which were not detected by Pair 1-based LFIA, were 2.84 × 10^4^ copies/mL, 5.58 × 10^3^ copies/mL, and 7.85 × 10^3^ copies/mL, respectively. These three specimens contained the lowest viral load among all patient specimens. Therefore, we confirmed that the detection limit of the Pair 1-based LFIA was approximately 1 × 10^5^ copies/mL (patient no. 11, viral load = 1.31 × 10^5^ copies/mL), and the Pair 2-based LFIA could detect virus concentrations as low as ~3 × 10^7^ copies/mL. As we previously demonstrated, our monoclonal antibodies employed in LFIAs recognized conserved sequences of SARS-CoV-2 that differed from those of SARS-CoV-2 variants, and recognized specific sequences that differed from other coronaviruses. Although the detection limit of LFIA was not as low as that of RT-qPCR, our LIFA could prove useful for rapid point-of-care testing for COVID-19 (e.g., home-testing and self-testing) in the face of rapidly spreading SARS-CoV-2 variants.

We also performed a specificity analysis of the LFIA to validate cross-reactivity with human coronaviruses and other respiratory pathogens causing clinical symptoms similar to those of SARS-CoV-2. Negative controls and their concentrations are listed in Experimental Section 2.7 and Figure S12. We observed no false-positive signals due to cross-reactivity with any of the 10 negative controls (Figure 5E), despite the relatively high concentration employed (10^4^ TCID50/reaction, tenfold higher than the detection limit of our LFIA device). These results indicate that our monoclonal antibody, developed from a conserved and specific short peptide of SARS-CoV-2, can successfully discriminate the SARS-CoV-2 spike antigen from other components of human coronaviruses and other respiratory pathogens.

## 4. Conclusions

SARS-CoV-2 has quickly evolved due to mutations at the gene and protein level. These mutations affect various properties of the virus, including transmissibility and antigenicity, and result in low sensitivity for detection of the SARS-CoV-2 antigen [18]. Herein, we identified conserved epitopes of SARS-CoV-2 NP for the development of specific monoclonal antibodies. Binding affinities and antibody pairs were investigated to develop a rapid biosensor. The sensitivity and specificity of the rapid biosensor were evaluated with recombinant NP, cultured viruses, clinical specimens, and other pathogens. The developed rapid biosensor could prove helpful for detecting SARS-CoV-2 NP under the circumstance of spreading SARS-CoV-2 variants, and the monoclonal antibody pair will be useful for developing other biosensors for SARS-CoV-2 variants.

## Data Availability

Amino acid sequences of SARS-CoV-2 NP (UniProtKB/Swiss-Prot: P0DTC9), SARS-CoV NP (UniProtKB/Swiss-Prot: P59595), and MERS-CoV NP (Uni-ProtKB/Swiss-Prot: K9N4V7) are available in the UniProt Knowledgebase (https://www.uniprot.org/uniprot/). The information of the frequencies of amino acid substitutions in SARS-CoV-2 NP is available in an open-source database tool (Nextstrain, https://nextstrain.org/ncov/gisaid/global).

## Supplementary Materials

The following supporting information can be downloaded at: https://www.mdpi.com/article/10.3390/v14020255/s1, Figure S1: Sequence alignment of SARS-CoV-2 NP antigen with SARS-CoV NP, MERS-CoV NP, 229E, and OC43; Figure S2: Schematic illustration of the SARS-CoV-2 viral components; Figure S3: Bar graph showing secondary screening results using indirect LFIA; Figure S4: Biolayer interferometry (BLI) results of monoclonal antibodies against antigenic peptides; Figure S5: Brightness and saturation adjusted images of the LFIA results for each of selected six pairs; Figure S6: Optimization of detection probe by adjusting the mixing ratio of 54G6 and 54G10; Figure S7: Brightness and saturation adjusted images of the LFIA results in the low concentration range for the sensitivity analysis; Figure S8: Brightness and saturation adjusted images of the LFIA results Detection sensitivity results of pair 1-based LFIA with clinical specimens; Figure S9: Detection sensitivity of the Pair 2-based LFIA with clinical specimens (*n* = 16); Figure S10: Pair 1- and Pair 2-based LFIA results using negative samples from healthy donors (*n* = 10); Figure S11: Results of RT-qPCR analysis with nasopharyngeal swabs from COVID-19 patients (*n* = 16) and healthy donors (*n* = 10); Figure S12: Analysis of the specificity of Pair 1- and Pair 2-based LFIA; Table S1: Detailed information for COVID-19 patients (*n* = 16) and healthy donors (*n* = 10).
