# Supplementary material for: Rapid Biosensor of SARS-CoV-2 Using Specific Monoclonal Antibodies Recognizing Conserved Nucleocapsid Protein Epitopes
Source: Viruses. 2022 Jan 27;14(2):255. doi: 10.3390/v14020255 (PMC8879994; doi:10.3390/v14020255)
Supplement: Supplementary file 1 [file viruses-14-00255-s001.zip › viruses-1531022-supplementary.pdf]

Rapid biosensor of SARS-CoV-2 using specific monoclonal antibodies recognizing conserved nucleocapsid protein epitopes

Jong-Hwan Lee[a], Yujin Jung[a], Sung-Kyun Lee[a], Jung Kim[a], Chang-Seop Lee[b], Soohyun Kim[a], Ji-Seon Lee[a], Nam-Hoon Kim[a, *], and Hong-Gi Kim[a,*]

[a] *Center for Convergent Research of Emerging Virus Infection, Korea Research Institute of Chemical Technology, Daejeon 34114, Republic of Korea*

[b] *Department of Internal Medicine, Jeonbuk National University Medical School, Jeonju, Jeollabuk-do 54896, Republic of Korea; Biomedical Research Institute of Jeonbuk National University Hospital, Jeonju, Jeollabuk-do 54907, Republic of Korea*

* Co-Corresponding authors: nhkim@krict.re.kr and Hong Gi Kim (tenork@krict.re.kr)

Supplementary Information

Supplementary Figures

Figure S1. Sequence alignment of SARS-CoV-2 NP antigen with SARS-CoV NP, MERS-CoV NP, 229E, and OC43.

Figure S2. Schematic illustration of the SARS-CoV-2 viral components.

Figure S3. Bar graph showing secondary screening results using indirect LFIA.

Figure S4. Biolayer interferometry (BLI) results of monoclonal antibodies against antigenic peptides.

Figure S5. Brightness and saturation adjusted images of the LFIA results for each of selected six pairs.

Figure S6. Optimization of detection probe by adjusting the mixing ratio of 54G6 and 54G10.

Figure S7. Brightness and saturation adjusted images of the LFIA results in the low concentration range for the sensitivity analysis.

Figure S8. Brightness and saturation adjusted images of the LFIA results Detection sensitivity results of pair 1-based LFIA with clinical specimens.

Figure S9. Detection sensitivity of the Pair 2-based LFIA with clinical specimens (n = 16).

Figure S10. Pair 1- and Pair 2-based LFIA results using negative samples from healthy donors (n = 10).

Figure S11. Results of RT-qPCR analysis with nasopharyngeal swabs from COVID-19 patients (n = 16) and healthy donors (n = 10).

Figure S12. Analysis of the specificity of Pair 1- and Pair 2-based LFIA.

Supplementary Tables

Table S1. Detailed information for COVID-19 patients (n = 16) and healthy donors (n = 10).

Figure S1

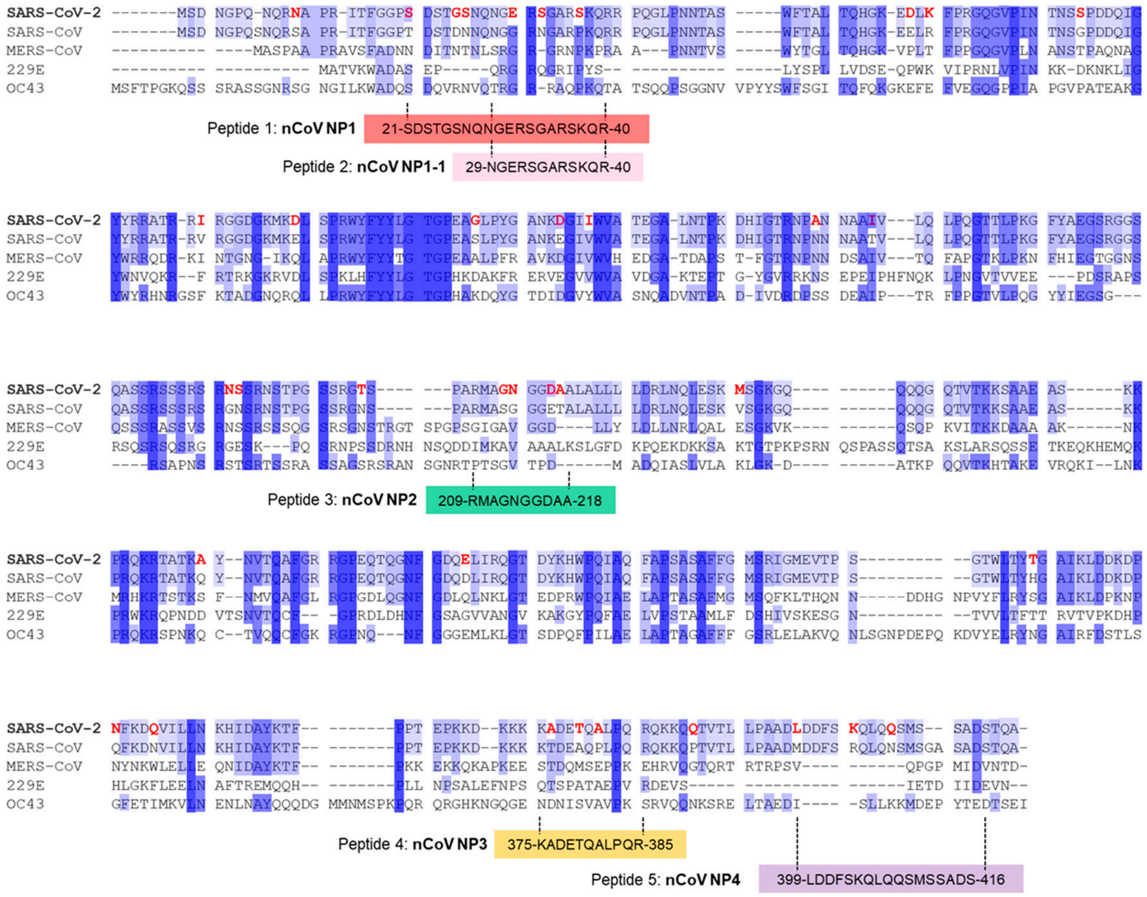

Figure S1. Sequence alignment of SARS-CoV-2 NP antigen with SARS-CoV NP, MERS-CoV NP, 229E, and OC43. SARS-CoV2 NP shares high sequence homology with NPs from SARS-CoV (~90% identity) and MERS-CoV (~50% identity). Five SARS-CoV-2-specific peptides with sequence specificity were selected as antigenic determinants.

Figure S2

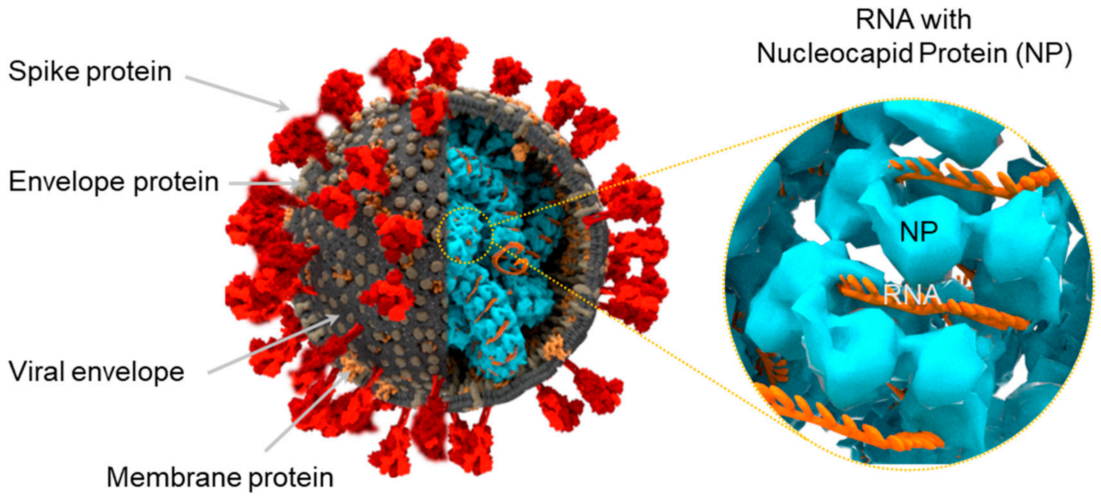

Figure S2. Schematic illustration of the SARS-CoV-2 viral components. NP (cyan) binds to single-stranded RNA (orange) in a helical capsid structure and plays a crucial role in packaging the viral genome.

Figure S3

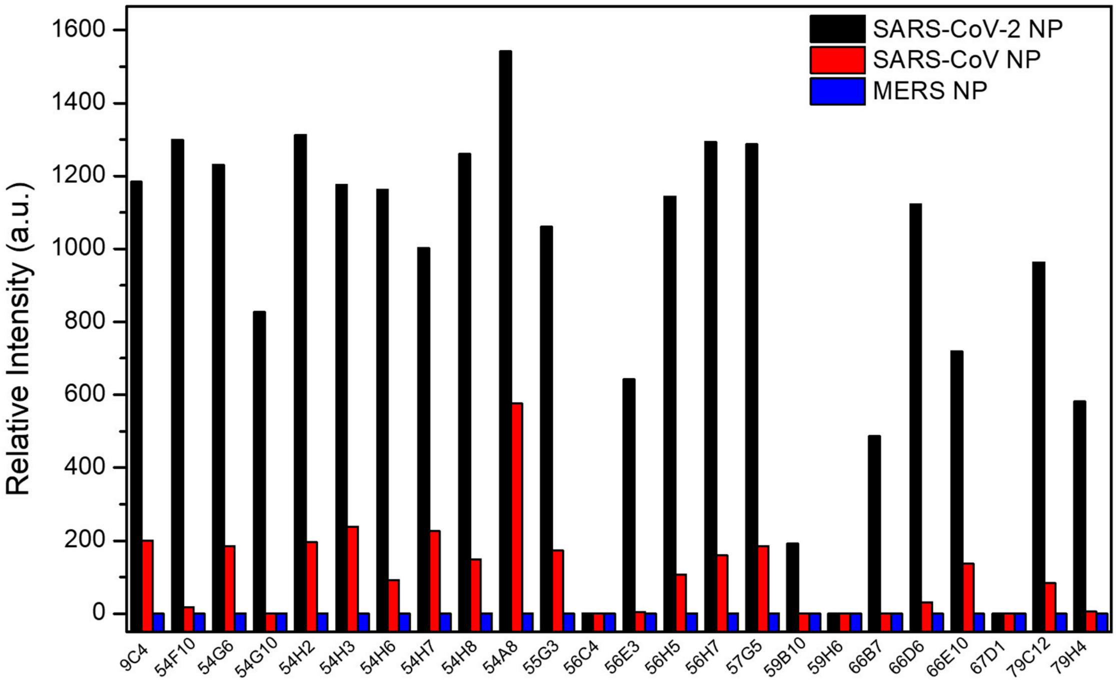

Figure S3. Bar graph showing secondary screening results using indirect LFIA. Three different NPs from SARS-CoV-2, SARS-CoV, and MERS-CoV were pre-immobilized onto a nitrocellulose membrane. The ability of the antibody to bind antigen was evaluated by flowing culture solution from a hybridoma containing a specific antibody to the previously immobilized antigen.

Figure S4

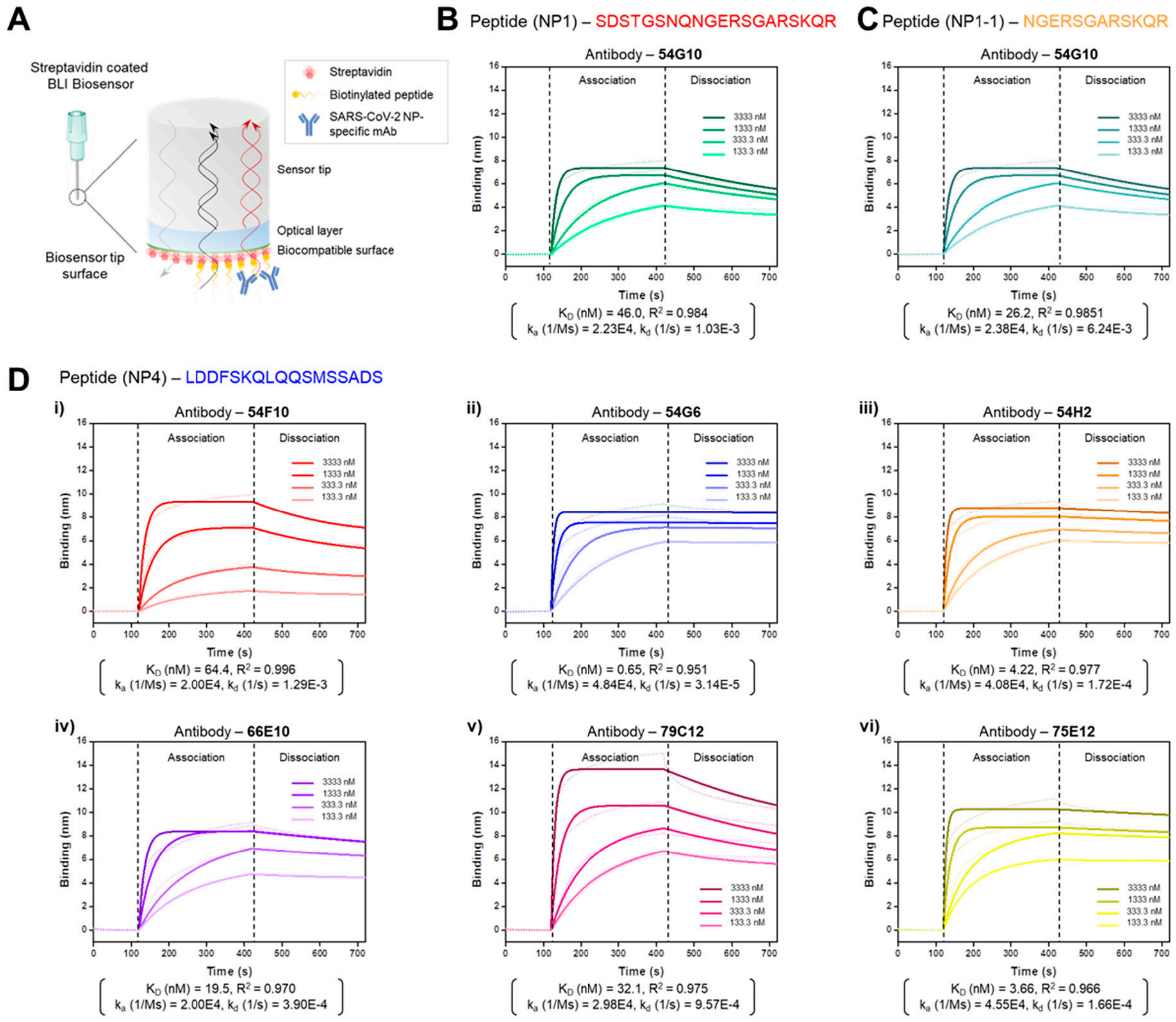

Figure S4. Biolayer interferometry (BLI) of monoclonal antibodies against antigenic peptides. A) Schematic illustration of the principle of BLI measurement based on antibody binding. B–D) Association and dissociation curves resulting from binding events between monoclonal antibodies and antigenic peptides. Real-time binding sensorgrams are represented as dotted lines, and their fitting curves are represented as solid lines. Binding constants were calculated from the fitting curves based on a 1:1 binding model.

Figure S5

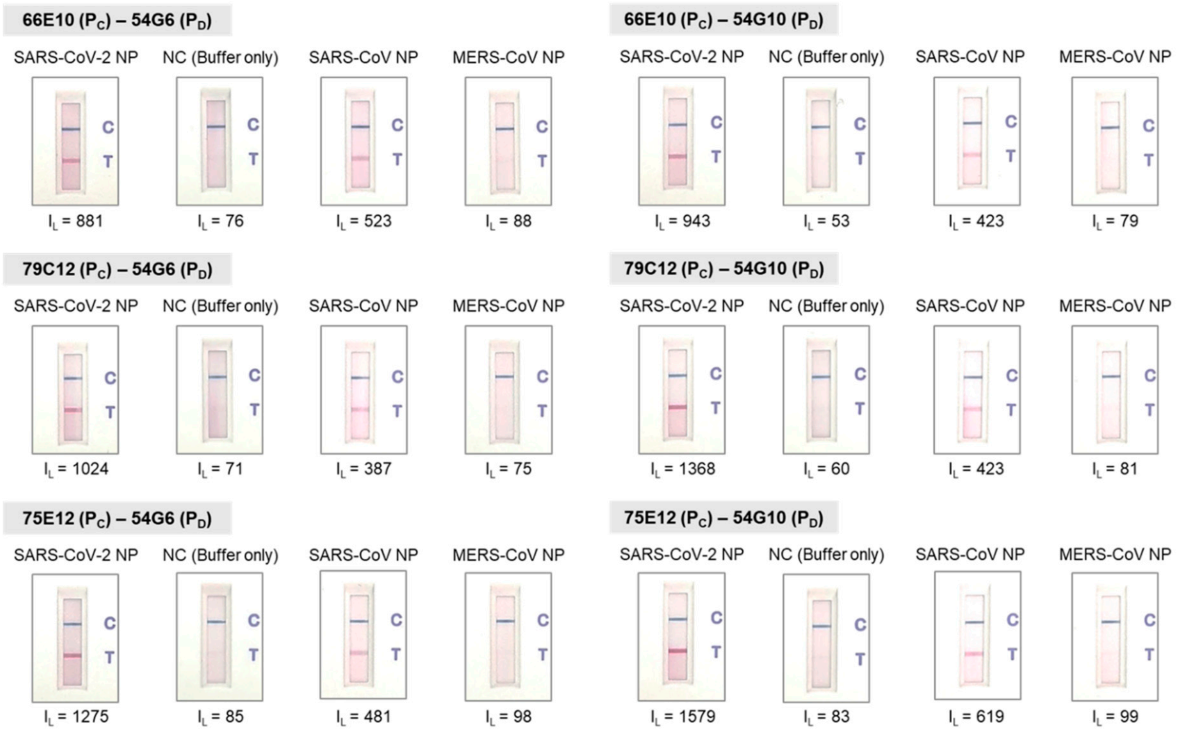

Figure S5. Brightness and saturation adjusted images of the LFIA results for each of the six pairs. Six pairs, namely 79C12(P_C)-54G10(P_D), 79C12(P_C)-54G6(P_D), 75E12(P_C)-54G10(P_D), 75E12(P_C)-54G6(P_D), 66E10(P_C)-54G10(P_D), and 66E10(P_C)-54G6(P_D), displayed remarkable detection sensitivity and are selected as sandwich pairs for detecting SARS-CoV-2.

Figure S6

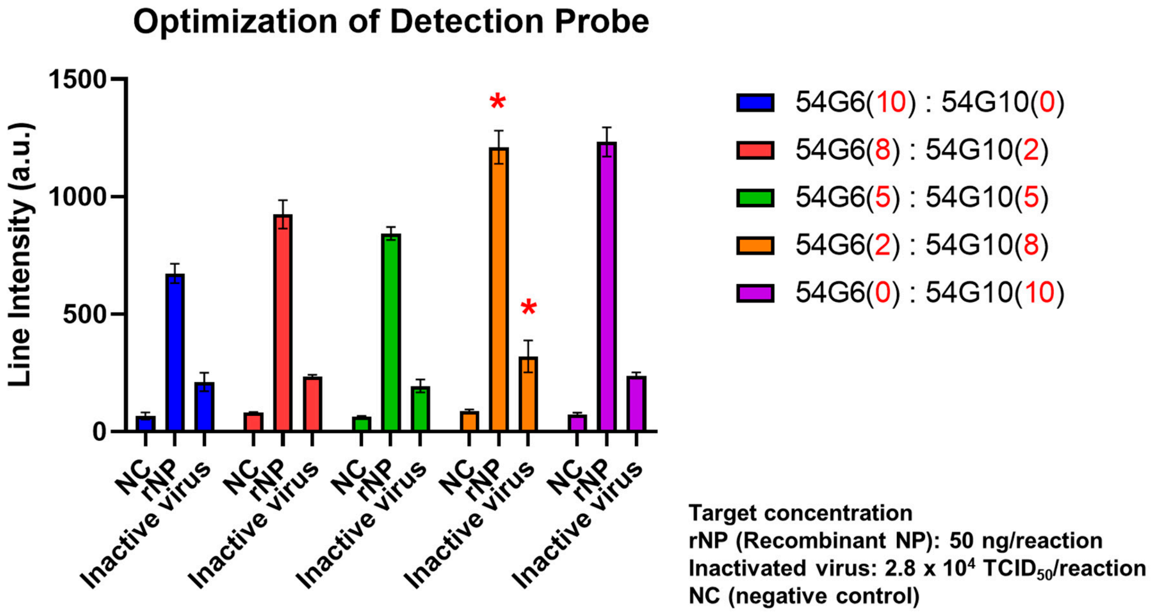

Figure S6. Optimization of detection probe by adjusting the mixing ratio of 54G6 and 54G10. Detection probes were optimized by mixing the selected detection probes (54G6 and 54G10) to improve detection sensitivity. Five different mixing ratios were assessed using 50 ng of recombinant SARS-CoV-2 antigen and 2.8 × 104 $TCID_{50}$ of inactivated viral samples. A 2:8 (v/v) ratio was identified as the optimal mixing ratio for detection probes.

Figure S7

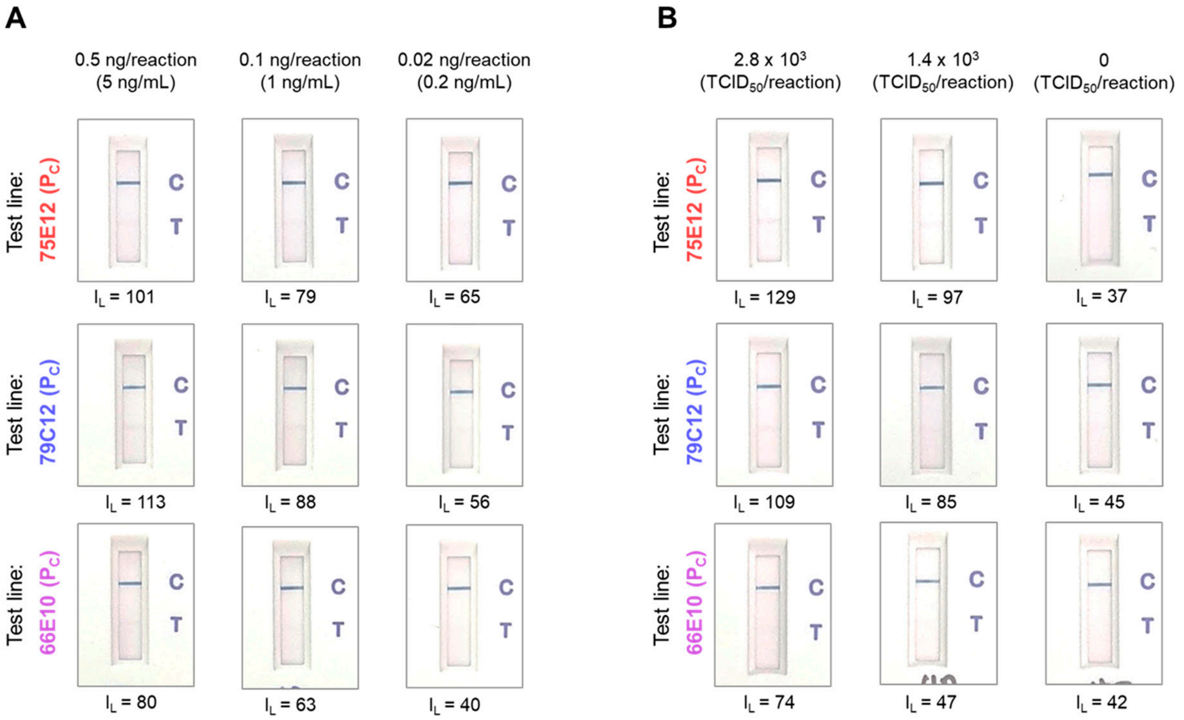

Figure S7. Brightness and saturation adjusted images of the LFIA results in the low concentration range for the sensitivity analysis. Three pairs of LFIAs, <Pair 1> 75E12 (P_C) – 54G6/54G10 (P_D), <Pair 2> 79C12 (P_C) – 54G6/54G10 (P_D), and <Pair 3> 66E10 (P_C) – 54G6/54G10 (P_D), are tested using serially diluted samples (concentration ranges – recombinant NP antigen: 0.5 ng to 0.02 ng antigen, viral sample: 5.6 x 10^3 $TCID_{50}$ to 1.4 x 10^3 $TCID_{50}$)

Figure S8

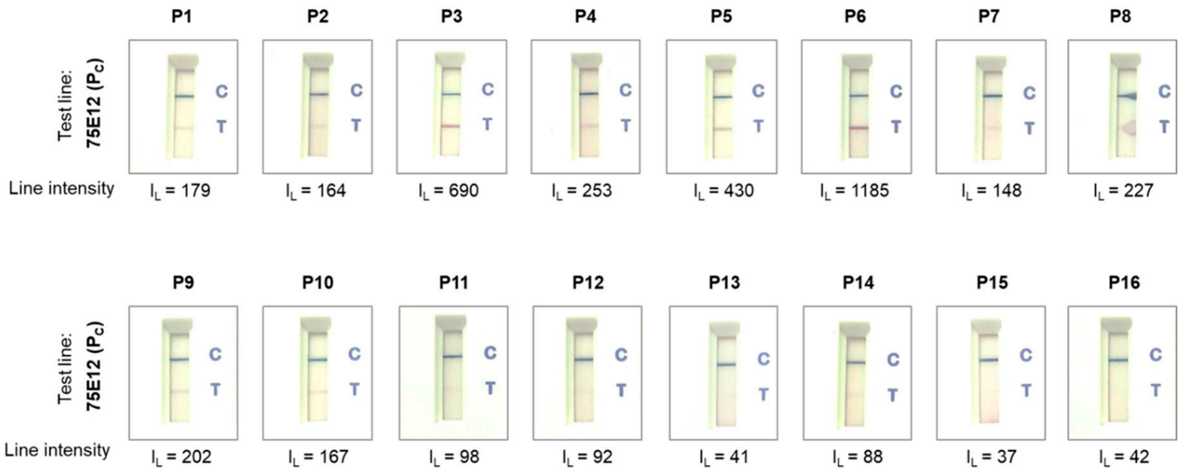

Figure S8. Brightness and saturation adjusted images of the LFIA results Detection sensitivity results of pair 1-based LFIA with clinical specimens. Nasopharyngeal swabs from COVID-19 patients (n=16) are applied to the LIFA device. After 15 min of sample loading, the results of COVID-19 infection are confirmed with the naked eye, and the intensities of the test lines are further analyzed with a portable analyzer (I_L: line intensity).

Figure S9

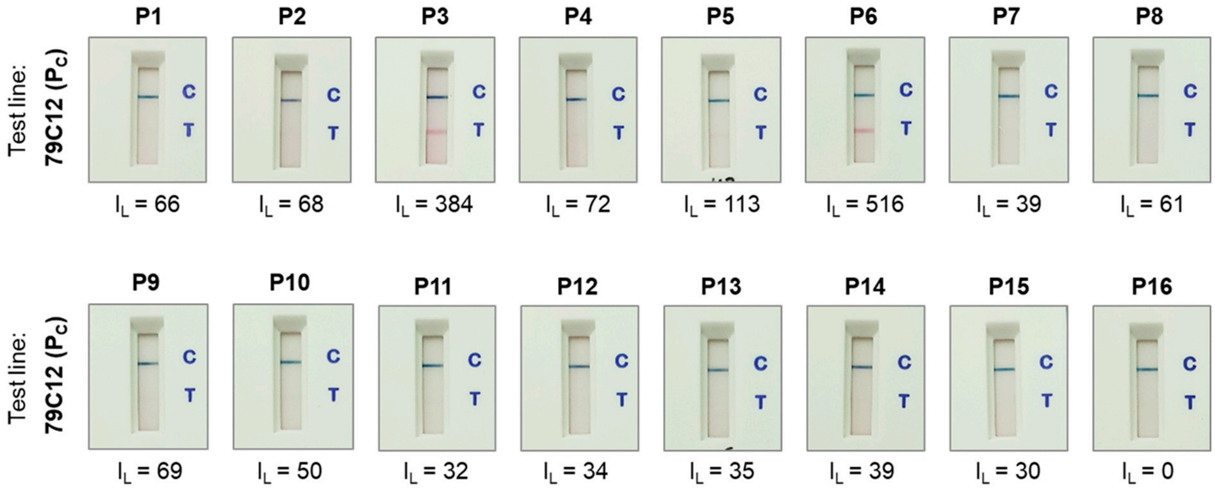

Figure S9. Detection sensitivity of the Pair 2-based LFIA with clinical specimens. Sixteen clinical specimens from COVID-19 patients are tested. The Pair 2-based LFIA detected 8/16 patient specimens but failed to detect specimens from patient No. 7, 10, 11, 12, 13, 14, 15, and 16.

Figure S10

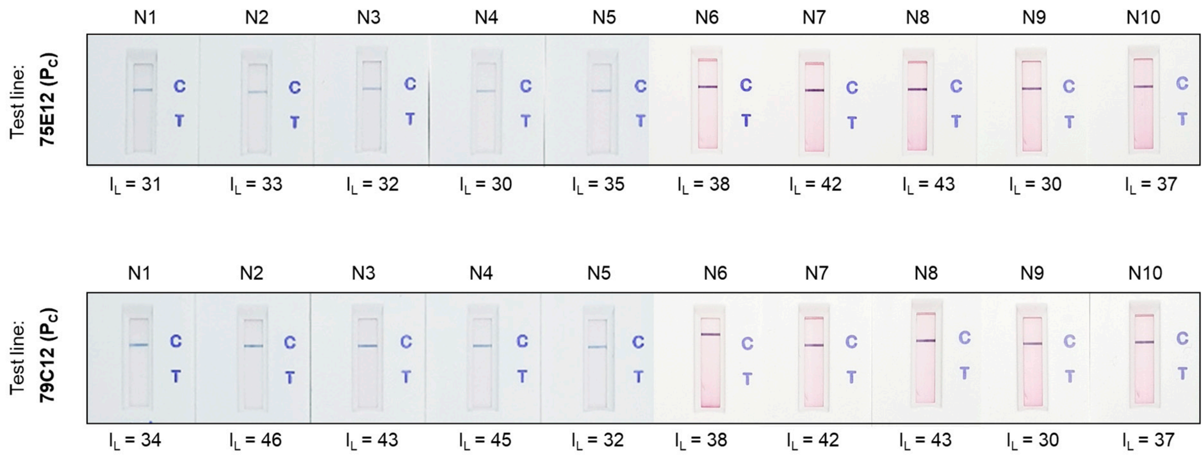

Figure S10. Pair 1- and Pair 2-based LFIA results using negative samples from healthy donors (n = 10). There were no false-positive signals for specimens from healthy donors for both Pair 1- and Pair 2-based LFIA.

Figure S11

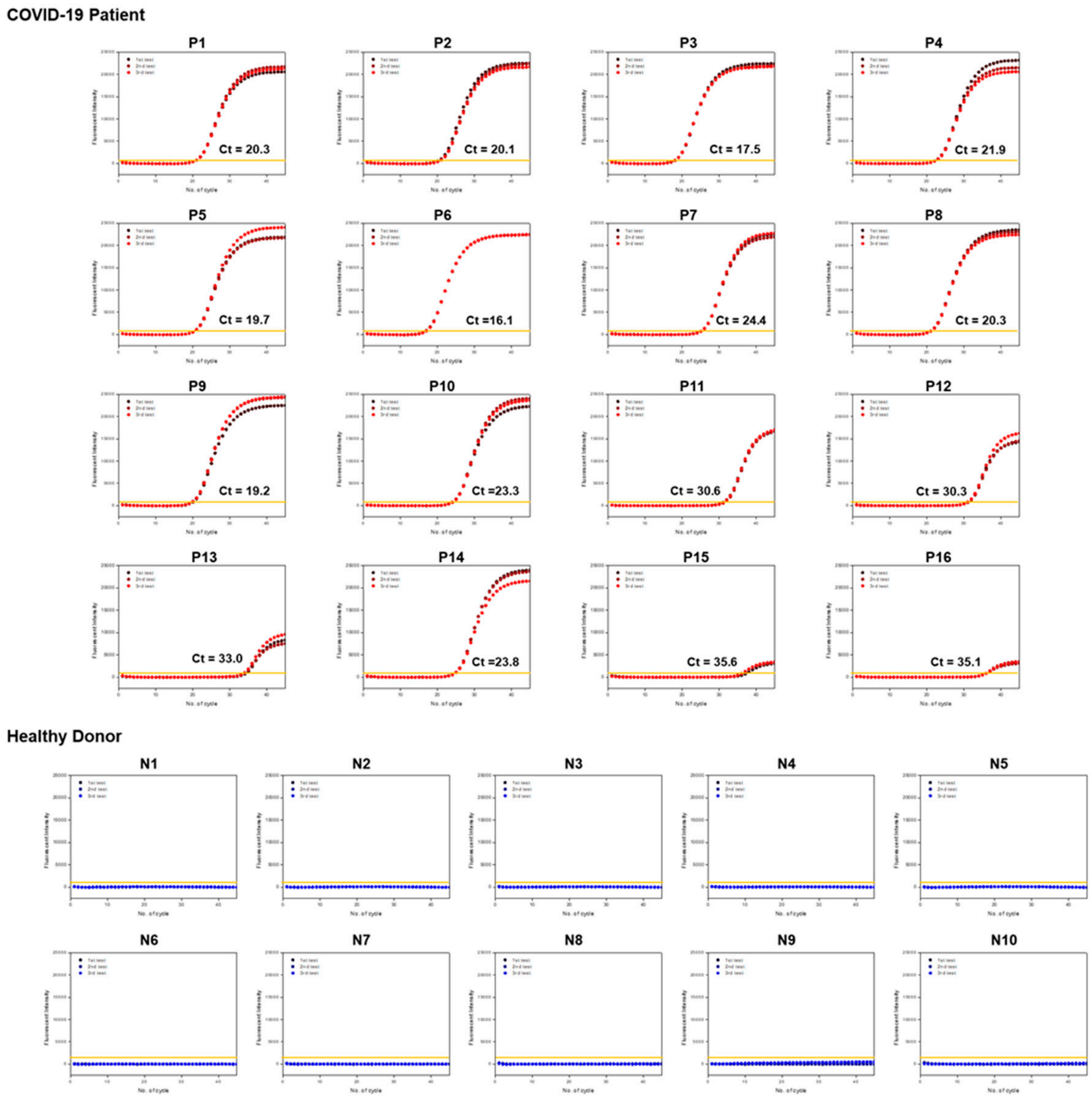

Figure S11. Results of RT-qPCR analysis with nasopharyngeal swabs from COVID-19 patients (n = 16) and healthy donors (n = 10). RT-qPCR was performed with specific primer-probe sets to amplify a specific gene (N-gene) of SARS-CoV-2.

Figure S12

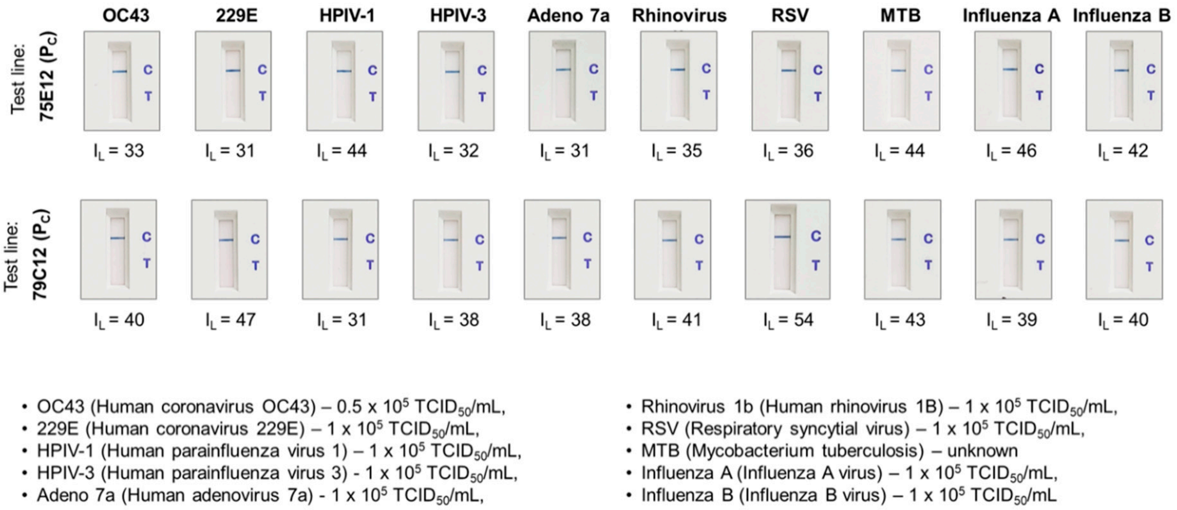

Figure S12. Analysis of the specificity of Pair 1- and Pair 2-based LFIA. Representative images of the LFIA specificity test results. Two human coronaviruses (OC43 and 229E) and other respiratory pathogens were tested, including human parainfluenza virus 1 (HPIV-1), human parainfluenza virus 3 (HPIV-3), human adenovirus 7a (Adeno 7a), human rhinovirus 1B (Rhinovirus 1b), human respiratory syncytial virus (RSV), and *Mycobacterium tuberculosis* (MTB). The concentration of the control virus sample was 10^6 $TCID_{50}$/reaction (5×10^5 $TCID_{50}$/reaction for OC43).

Table S1. Detailed information for COVID-19 patients (n = 16) and healthy donors (n = 10)

	Sample ID	Age	Gender	Matrix	Date of hospital admission
COVID-19 Patient	P1	54	Male	Nasopharyngeal swab	2020. 12. 08
	P2	59	Female	Nasopharyngeal swab	2020. 12. 18
	P3	33	Female	Nasopharyngeal swab	2020. 12. 19
	P4	67	Male	Nasopharyngeal swab	2020. 12. 24
	P5	61	Female	Nasopharyngeal swab	2020. 12. 24
	P6	44	Female	Nasopharyngeal swab	2021. 02. 13
	P7	83	Male	Nasopharyngeal swab	2021. 02. 16
	P8	78	Female	Nasopharyngeal swab	2021. 02. 16
	P9	35	Female	Nasopharyngeal swab	2021. 02. 26
	P10	30	Female	Nasopharyngeal swab	2021. 02. 28
	P11	65	Female	Nasopharyngeal swab	2021. 03. 02
	P12	23	Female	Nasopharyngeal swab	2021. 02. 28
	P13	22	Female	Nasopharyngeal swab	2021. 02. 26
	P14	32	Female	Nasopharyngeal swab	2021. 02. 26
	P15	68	Female	Nasopharyngeal swab	2021. 01. 06
	P16	51	Male	Nasopharyngeal swab	2021. 01. 12
Healthy controls	N1	59	Female	Nasopharyngeal swab	-
	N2	47	Male	Nasopharyngeal swab	-
	N3	63	Female	Nasopharyngeal swab	-
	N4	29	Male	Nasopharyngeal swab	-
	N5	41	Male	Nasopharyngeal swab	-
	N6	66	Female	Nasopharyngeal swab	-
	N7	66	Male	Nasopharyngeal swab	-
	N8	50	Male	Nasopharyngeal swab	-
	N9	34	Male	Nasopharyngeal swab	-
	N10	29	Male	Nasopharyngeal swab	-
